# Monochelic Versus Telechelic Poly(Methyl Methacrylate) as a Matrix for Photoluminescent Nanocomposites with Quantum Dots

**DOI:** 10.3390/molecules26144131

**Published:** 2021-07-07

**Authors:** Anastasija D. Jablanovic, Marianna Z. Bekanova, Ekaterina A. Litmanovich, Oleg N. Karpov, Miron A. Bugakov, George A. Shandryuk, Alexander A. Ezhov, Raisa V. Talroze, Elena V. Chernikova

**Affiliations:** 1 Faculty of Materials Science, M.V. Lomonosov Moscow State University, Leninskie Gory 1-73, 119991 Moscow, Russia; chlorophyta98@gmail.com; 2 Faculty of Chemistry, M.V. Lomonosov Moscow State University, Leninskie Gory 1-3, 119991 Moscow, Russia; bkmarianna@yandex.ru (M.Z.B.); elitmanovich@yandex.ru (E.A.L.); bugakov.miron@gmail.com (M.A.B.); 3 A.V. Topchiev Institute of Petrochemical Synthesis, Russian Academy of Sciences, Leninsky Prospect 29, 119991 Moscow, Russia; o-karpov777@mail.ru (O.N.K.); gosha@ips.ac.ru (G.A.S.); alexander-eshov@yandex.ru (A.A.E.); 4 Faculty of Physics, M.V. Lomonosov Moscow State University, Leninskie Gory 1-2, 119991 Moscow, Russia

**Keywords:** quantum dots, nanocomposites, RAFT polymerization, photoluminescence, dynamic light scattering

## Abstract

Nanocomposites based on CdSe or CdSe/ZnS quantum dots (QDs) and poly(methyl methacrylate) (PMMA) of different molecular weights and functionality were synthesized by ligand exchange of oleic acid with RAFT-based PMMA. The successful ligand exchange was confirmed by dynamic light scattering in combination with the approach “macromolecules—ghosts” and transmission electron microscopy. Comparative study of mono- and telechelics of PMMA revealed the similarities and differences in their behavior in formation of complexes with QDs and the optical properties of the corresponding nanocomposites. Telechelics exhibited higher efficiency in the complex formation and seemed to be promising candidates for the construction of devices based on QDs and polymer matrix for optical applications.

## 1. Introduction

Nowadays, semiconductor quantum dots (QDs) based on CdSe, ZnSe, CdTe, CdS, ZnS, InAs, InP, etc. attract a great deal of interest due to their unique size-dependent chemical and optical properties, and consequently, a wide range of their possible applications in optical, non-linear optical, and electronic devices and biomedicine [1,2,3,4,5,6]. Construction of devices based on QDs as an active medium requires rather often their embedding into a polymer matrix that may give rise to novel optical properties different from those of pure QDs and the matrix, as happens in the case of liquid crystal polymers [7]. However, the embedding of QDs into the matrix is accompanied by the suppression of their mobility. In this case, the photoluminescent properties of QDs will depend on their size and order in the matrix, the accessibility of reactive groups at their surface, and the chemical structure of ligands stabilizing the surface of QDs [8,9,10].

QDs stabilized by low-molecular-weight ligands (e.g., oleic acid, trioctylphosphine (TOP), etc.) can be embedded in the polymer matrix via physical methods, such as mixing solutions or melts of the polymers and QDs, either with further removal of the solvent by evaporation, precipitation, and electro-spinning; by extrusion; by pre-polymerization techniques; or by encapsulation [11,12,13,14,15,16,17,18,19]. Despite the visible simplicity of these approaches, QDs are dispersed mostly non-uniformly in the polymer matrix apart from encapsulation techniques, resulting in the macrophase separation and worsening of optical properties of QDs and mechanical properties of the matrix. Additionally, the mentioned approaches suffer from multi-stage processing and relatively high cost.

Thus, the direct stabilization of QDs by polymers through chemical binding with the reactive groups on the surface of QDs is more promising, as it obviates difficulties mentioned above. In this case, macromolecules should contain side or terminal functional groups such as carboxyl, pyridine, amine, thiol, etc., able to form ionic or covalent bonds with corresponding reactive groups located at the surface of QDs [20,21,22]. These techniques include ligand exchange, grafting from, and grafting to approaches, and they cover a wide range of polymers of different chemistry and molecular architecture [23,24,25,26]. The control over amount and distribution of the functional side groups within the macromolecule, the molecular architecture of the chain, and the chemical nature of the terminal functional groups is governed by the appropriate choice of the synthetic route. Among known polymerization techniques, reversible addition–fragmentation chain transfer (RAFT) polymerization seems the most versatile technique to solve these tasks [27,28,29,30,31,32]. Apart from the tolerance to the functional groups of the reagents and mild conditions of realization, RAFT polymerization provides the controlled synthesis of macromolecules of given functionality with desired molecular architecture like living ionic polymerization [33,34,35]. One of the interesting examples of the creation of polymer–QD nanocomposites using the grafting from approach is the use of QDs themselves as a photocatalyst during RAFT polymerization [36].

The RAFT technique has already been applied to the synthesis of polymers with appropriate functional side groups able to stabilize QDs in the polymer matrix [33]. For example, a block copolymer PS-b-P(S-co-pyren-1-yl) with fluorescent properties was synthesized via RAFT polymerization and used as a donor component in the fluorophores pair copolymer/CdTe QDs to experience Förster resonance energy transfer with an efficiency of 48%, being an efficient antenna system for light harvesting applications [34]. Surface-initiated RAFT polymerization was used in the synthesis of poly(poly(ethylene glycol) methacrylate) grafted to CdTe QDs, followed by bioconjugation and post-functionalization by adenosine. The photoluminescence properties of the synthesized hybrids revealed their great potential for application as optimal materials in biomedicine. [35]. Nanocomposites based on CdSe/ZnS core-shell QDs encapsulated in poly(styrene-co-maleic anhydride) prepared by RAFT copolymerization and cross-linked with aminopropyl-terminated polydimethylsiloxane were fabricated for application in optoelectronic devices. This approach gave rise to a nanocomposite with uniform dispersion of QDs (QD concentration up to ~30 wt%) and superior optical properties [37]. 

However, the binding of nanoparticles with terminal groups of the polymer seems more attractive, as in this case the length of the macromolecules may control the distance between QDs. This approach was first applied to RAFT-based acrylic cholesteric copolymer containing one thiol-terminal group and capable of binding the surface of Au nanoparticles (Au NPs) [38]. The authors succeeded in maintaining the liquid crystalline ordering of a matrix with a high concentration of nanoparticles. This idea was further developed [39] for the synthesis of thiol-functionalized liquid crystal homopolymers and random copolymers based on acrylates containing mesogenic groups. Finally, it was applied to RAFT-based glass dielectric polymers, as they are suitable for the design of devices for optical and non-linear optical applications [40,41]. Thus, the binding of poly(methyl methacrylate) (PMMA) samples that contain either one (monochelic) or two (telechelic) terminal carboxylic and/or thiol groups with the CdSe QDs was studied by dynamic light scattering (DLS) using an approach of the “macromolecules ghosts” [42]. In this research we discovered the correlation between end-functionality, molecular weight of the polymer, and the size of the complexes. 

Monochelic polystyrenes with terminal thiol group of different molecular masses were used for designing nanocomposites based on CdSe QDs and Au nanoparticles (NPs). It was demonstrated that the photoluminescence of QDs in the sol grew during continuous irradiation but were reduced during the “light switching off–switching on” process. Simultaneously, upon the addition of Au NPs, the photoluminescence of QDs in the sols changed insignificantly [40]. The size and morphology of the complexes of monochelic polystyrene–QDs in the sols and in the solid films were studied and several possible models of the complexes have been developed [43]. The similar monochelic polystyrenes were used to fabricate composites with CdSe/ZnS core-shell QDs and Au NPs. The photoluminescence of QDs in nanocomposites decreased after embedding of Au NPs due to combined inner filter effects and the influence of the dielectric properties of the medium surrounding Au NPs embedded in polystyrene–CdSe/ZnS composites [44].

The monochelic amine-terminated PMMA with various molecular masses was used to fabricate highly luminescent and transparent nanocomposites based on CdSe/ZnS QDs [45]. The mentioned nanocomposite with oligomeric PMMA chains (M_w_~2 kDa) had high quantum efficiency and transmittance even at high QD concentration (49 wt%), which was attributed to the enhanced passivation of the QD surface due to the high grafting density of the polymeric ligands and more uniform dispersion of QDs. 

Thiol-terminated poly(methyl methacrylate-block-glycidyl methacrylate) was used to produce nanocomposites with CdSe/ZnS core-shell QDs [41]. The authors proposed a facile and effective passivation method to enhance the photochemical stability of QDs using polymeric double shell structures: highly transparent PMMA outer-shell and oxidation-protective cross-linked inner shell. The latter was obtained by the cross-linking reaction of epoxides in a poly(glycidyl methacrylate) block. The resulting QDs exhibited exceptional tolerance to heat and oxidants when dispersed in organic solvents or QD nanocomposite films. 

The major goal of our present research was to reveal the influence of the number of the functional end-groups in polymer macromolecules (mono- and telechelics), as well as the type of QDs on the formation and properties of nanocomposites. We chose PMMA as a polymer matrix, which is well-known to have terminal groups of different kinds when RAFT polymerization is applied [32,46] and to have the general optical properties of PMMA films that provide pretty high optical transparency.

## 2. Results and Discussion

### 2.1. Formation of PMMA Mono- and Telechelic Complexes with CdSe and CdSe/ZnS Quantum Dots

**Scheme 1:**
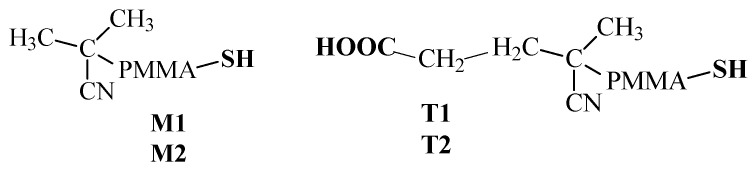
Chemical structure of monochelic and telechelic PMMA.

The detailed procedure of the synthesis of PMMA is described in the Section 3. Here we define thiol-terminated PMMA as monochelic (see Scheme 1, **M1**, **M2**) and PMMA containing both carboxyl and thiol end-groups as telechelic (**T1** and **T2**).

According to our previous research [41], CdSe spherical QDs do not really interact with PMMA, which contains no appropriate functional groups, and unbound PMMA does not influence the size distribution function of QDs. In contrast, mono- and telechelics of PMMA containing carboxyl and/or thiol end-group can form nanocomposites with CdSe QDs after the mixing of toluene solution of PMMA and toluene sol of QDs through ligand exchange between oleic acid stabilizing QDs and end-functionalized PMMA. Our previous studies have shown that the interaction of macromolecules and active centers on the surface of CdSe spherical QDs, i.e., the sites at the QD surface at which reaction with a macromolecule end-group occurs, can be followed by DLS in toluene [42]. The general approach called as “macromolecules—ghosts” is based on the isorefractivity of PMMA (n_D_^20^ = 1.492) and toluene (n_D_^20^ = 1.497), which makes free PMMA macromolecules invisible in toluene solution. However, when PMMA forms a complex with QDs that have a different refractive index from toluene, the complex becomes visible, and one can measure the diffusion coefficient of this complex and its hydrodynamic radius, while free (unbound) PMMA remains invisible to detection. 

It is reasonable to assume that mono- and telechelics of PMMA will keep this ability to interact with CdSe non-spherical QDs and CdSe/ZnS core-shell QDs surface through ligand exchange. According to the DLS data, the average hydrodynamic radius of both types of QDs is 5 ± 1 nm. In all the experiments, we used a constant concentration of PMMA equal to 5 mg/mL and varied the concentration of QDs in sol in the range of 0.085–2.4 mg/mL, similar to previously studied systems [42]. The given amount of sol of QDs was mixed with solution of PMMA in toluene; the composition of the resulting mixture was characterized by Z, defined as the ratio of the weight concentrations of QDs and PMMA (Z = C_QDs_/C_PMMA_). Based on our previous results, we expected that monochelic PMMA would interact with QDs via one end-group, while telechelic PMMA would interact via both end-groups. 

**Figure 1:**
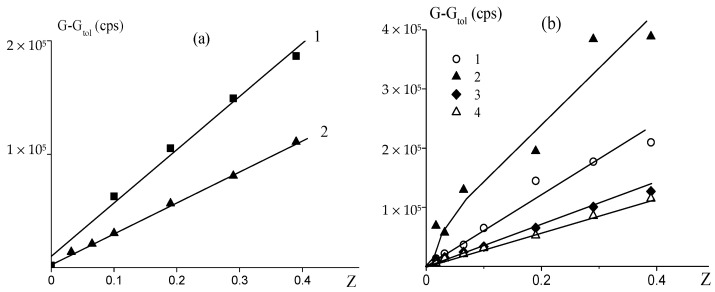
The scattering intensity as a function of the composition Z of the mixtures of non-functionalized PMMA and (1) CdSe non-spherical QDs or (2) CdSe/ZnS QDs (**a**); (1) monochelic **M1**—CdSe QDs, (2) telechelic **T2**—CdSe QDs, (3) telechelic **T1**—CdSe/ZnS QDs, (4) telechelic **T2**—CdSe/ZnS QDs (**b**). Here and below, toluene was used as a solvent, C_PMMA_ = 5 mg/mL, T = 25 °C.

The dependences show linear growth of scattering intensity with the increase of Z, i.e., of QDs concentration in the mixture for both types of QDs in the presence of non-functionalized PMMA (Figure 1a). The different slope of the dependences is caused by various polarizabilities of CdSe non-spherical QDs and CdSe/ZnS core-shell QDs, and as a consequence, the different optical contrast in toluene medium. The scattering intensity of mixtures of CdSe non-spherical QDs is similar to what was discovered previously for spherical QDs [42]. In both systems the value of the hydrodynamic radius of scattering particles remained constant and equal to the hydrodynamic radius of QDs 5 ± 1 nm. Both plots presented in Figure 1a can be used for comparison with other investigated systems (Figure 1b), as the Z scale is the same, i.e., the same Z values correspond to the same QDs concentrations.

Figure 1b presents the typical dependences of scattering intensity as a function of the composition Z for the systems of monochelic and telechelic PMMA with CdSe non-spherical QDs (curves 1, 2) and CdSe/ZnS core-shell QDs (curves 3, 4). It can be seen that in all the cases the scattering intensity progressively grows with Z, i.e., with the rise of the content of QDs in the mixture. Comparison of Figure 1a,b shows the similar values of scattering intensity for unbound CdSe QDs (Figure 1a, curve 1) and monochelic M1—CdSe QDs (Figure 1b, curve 1) and of unbound CdSe/ZnS QDs (Figure 1a, curve 2) with telechelics **T1** or **T2** and CdSe/ZnS QDs (Figure 1b, curves 3 and 4).

**Figure 2:**
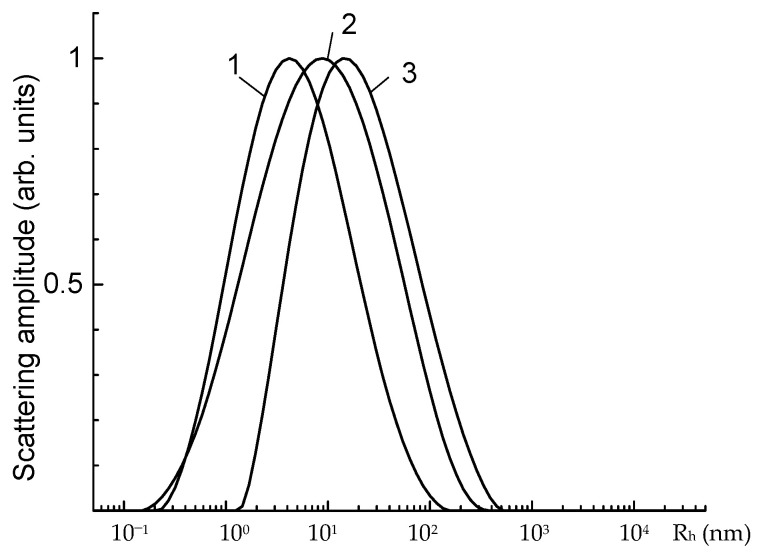
Hydrodynamic radius distribution of scattering particles for the CdSe QDs toluene sol (1), mixture of CdSe QDs—M1 with composition Z of mixture 0.1 (2) and 0.065 (3).

**Figure 3:**
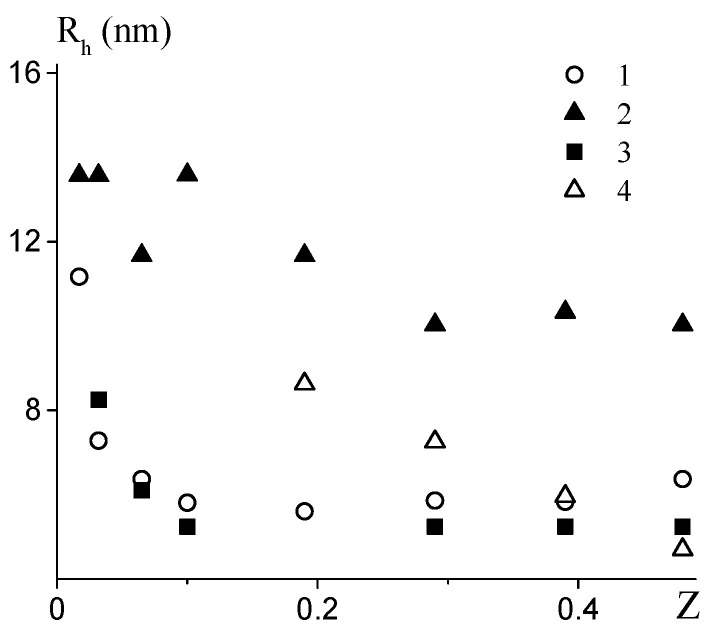
Hydrodynamic radius R_h_ as a function of composition Z for the toluene sols of (1) monochelic **M1**—CdSe QDs, (2) telechelic **T2**—CdSe QDs, (3) telechelic **T1**—CdSe/ZnS QDs, and (4) telechelic **T2**—CdSe/ZnS QDs.

Independent of the close values between the scattering intensity as a function of the composition Z (Figure 1a and Figure 3b), the hydrodynamic radius of the QDs interacting with a polymer functional group increases with the decrease in Z. As an example, the hydrodynamic radius distribution of scattering particles is given in Figure 2 for CdSe QDs and **M1**—CdSe non-spherical QDs at Z = 0.1 and 0.065. One can see that the average hydrodynamic radius R_h_ of scattering particles varies for the pristine QDs and the mixtures of monochelics and QDs. The decrease of Z, i.e., in excess of weight content of PMMA with respect to QDs, results in the increases of the average hydrodynamic radius R_h_. Taking into account the isorefractivity of PMMA and toluene, this indicates that that macromolecules form complexes with nanoparticles, which are visible for DLS.

In contrast, the scattering intensities for **T2** and non-spherical CdSe QDs are about twice-higher (Figure 1b, curve 2) than those observed in other systems. The comparison of mixtures of the same composition of telechelic PMMA **T2** with non-spherical CdSe QDs and spherical CdSe QDs [42] shows that the scattering intensities as a function of Z in both cases are characterized by S-shape, but for non-spherical CdSe QDs, the scattering intensity is higher than for spherical ones. 

The typical dependences of the hydrodynamic radius R_h_ of scattering particles for complexes of **M1**, **T1**, **T2** with CdSe, and CdSe/ZnS QDs as function of Z are presented in Figure 3. In all the cases, at low values of Z, the size of scattering particles exceeds the size of QDs, which means that the ligand exchange occurs, resulting in the formation of the complexes of QDs with mono- and telechelic PMMA. As we have shown previously [42], the plateau, which is expected on the plot in this region, can be achieved rarely due to the loss of the aggregative stability of QDs at their low concentrations. With the increase in Z values, i.e., at high content of QDs, R_h_ value should tend to the size of QDs (curves 1, 3, 4).

**Figure 4:**
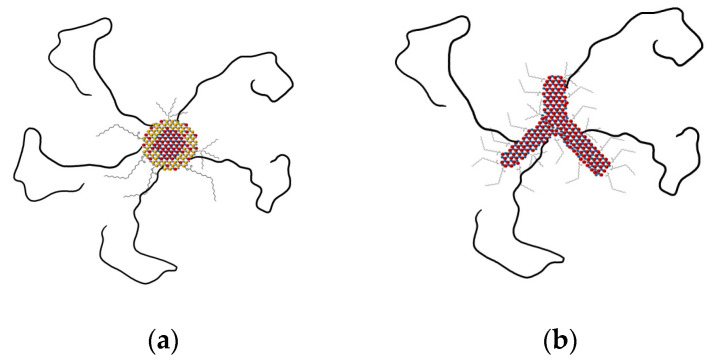
Schematic presentation of spherical core-shell CdSe/ZnS (**a**) and non-spherical CdSe (**b**) QDs chemically bound with macromolecules.

The changes of the hydrodynamic radius of the mixtures of **M1** with CdSe (Figure 3, curve 1) and **T1** with CdSe/ZnS (Figure 3, curve 3) at different compositions are similar. Combining the results presented in Figure 1 and Figure 3, it may be supposed that both types of macromolecules are attached to the QD via one end-group. Schematically it is shown in Figure 4 for spherical core-shell QD (a) and nonspherical CdSe QD (b).

This conclusion is evident for monochelic PMMA, which contains one functional end-group per chain (Figure 3, curve 1). In the case of telechelic PMMA **T1** with COOH and SH end-groups, the use of core-shell CdSe/ZnS QDs leads to the interaction of the macromolecule only with one group at conditions corresponding to DLS measurements (Figure 3, curve 3). This unexpected result may indicate that COOH and SH groups have different binding ability with the surface of core-shell QDs. This assumption correlates with the results of the thermodynamic studies of the SH and COOH binding ability with ZnS described in [47]. The increase of molecular weight (MW) of telechelic PMMA **T2** results in the expected growth of the size of the complex (Figure 3, curve 4).

**Figure 5:**
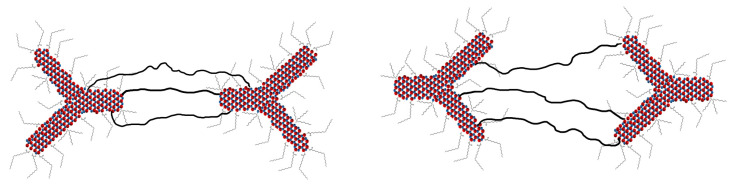
Possible schematic presentations of non-spherical CdSe QD bound with telechelic PMMA **T2**.

In contrast, the complex of **T2** and CdSe non-spherical QDs exhibits different behavior at high Z values. One can see the tendency of R_h_ to reach double the size of QDs (Figure 3, curve 2). It may be supposed that at high content of QDs, one macromolecule of **T2** binds to two CdSe non-spherical QDs, thus causing the double increase in the scattering intensity visible in Figure 1b, curve 2. This difference discovered for CdSe QDs may be caused by their non-spherical shape, which may restrict to some extent the binding of macromolecules by both end-groups to one QD and provoke binding of telechelic PMMA to two neighboring QDs (Figure 5). It is important to note that these experiments were conducted in conditions of low concentrations of PMMA and QDs in solution required for DLS experiments. 

The continuous increase of the concentrations of both PMMA and QDs may result in the formation of a network due to the intermolecular binding of telechelics and QDs.

The macromolecule attached to the surface of QDs may have different conformations, which depend on the MW of the polymer, the density of the chains attached to the surface, and the thermodynamic quality of the solvent. According to our previous study, the PMMA macromolecules with MW above 7 kDa bound in complex with QDs in toluene have the conformation of the swollen coil or “mushroom” [42]. It may be supposed that this conformation is kept regardless of the shape and type of QDs. 

Thus, we have shown several examples that mono- and telechelic PMMA can form complexes with CdSe non-spherical QDs and CdSe/ZnS core-shell QDs.

### 2.2. Morphology and Molecular Structure of Complexes of QDs with PMMA

**Figure 6:**
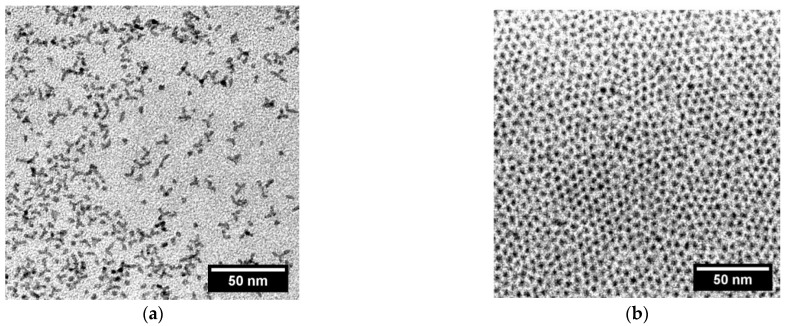

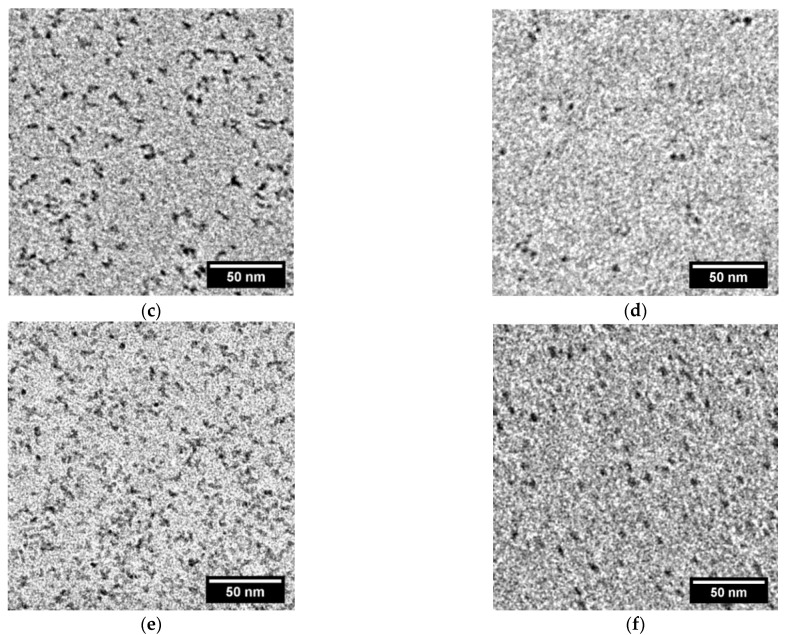
TEM microphotographs of dried sols of CdSe (**a**) and CdSe/ZnS QDs (**b**) and monochelic PMMA **M1** and telechelic PMMA **T1** and QDs at Z = 0.1: **M1**—CdSe (**c**), **M1**—CdSe/ZnS (**d**), **T1**—CdSe (**e**), **T1**—CdSe/ZnS (**f**).

To analyze the morphology of complexes, we used transmission electron microscopy (TEM) measurements. The corresponding microphotographs are presented in Figure 6. Dried sols of QDs demonstrate the shape difference of CdSe (a) and CdSe/ZnS (b). In the absence of PMMA, QDs are more densely packed than in the presence of polymers. 

When one considers the morphology of polymer samples with embedded QDs (Figure 6c–f), it is necessary to be aware that systems under study should contain both polymer-bound and non-bound QDs. To estimate the amount of bound QDs, we purified the sols from unbound “free” QDs via precipitation in hexane. Mono- and telechelics and their complexes precipitate, while QDs remain in stock solution colorizing it in red. After that, the stock solution was separated, and nanocomposites were dissolved in toluene and then re-precipitated in hexane. This procedure was repeated to achieve colorless stock solution. The weight fractions of QDs bound and non-bound with PMMA in toluene sols were determined from calibration curves. The known amount of nanocomposites was dissolved in toluene (2.5 mg/mL), and the absorption spectra of solutions were recorded. The results of the analysis are summarized in Table 1.

There is quite a reasonable problem regarding the type of bonds providing the formation of chemically bound nanocomposites. As shown in [48], the synthesis of CdSe QDs described in the experimental part results in the stabilization of QDs due to the formation of oleic acid ligands in the form of cadmium oleate (1530 cm^−1^), whereas the stabilization of CdSe/ZnS QDs is reached via formation of both cadmium and zinc oleates (1530 and 1547 cm^−1^) at the QD’s surface [44].

**Figure 7:**
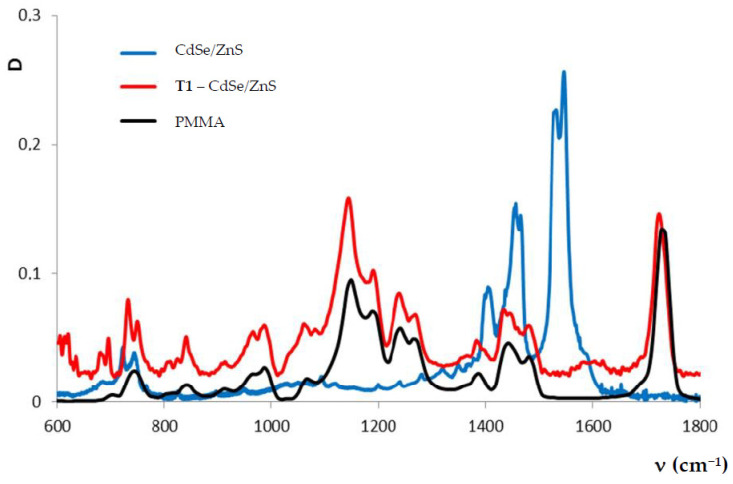
FTIR spectra in ATR mode of CdSe/ZnS QDs, PMMA, and T1/CdSe/ZnS QDs.

The process of the polymer ligands formation at the QD’s surface results from the ligand exchange when macromolecules substitute oleic acid. However, this process is reversible, and at the surface, one can see both polymer and oleate ligands. As an example, we compared FTIR spectra of conventional PMMA and **T1** PMMA—CdSe/ZnS QDs nanocomposite (Figure 7). In the FTIR spectrum of **T1**/CdSe/ZnS QDs, the intense band is observed at 1730 cm^−1^, which can be ascribed to stretching vibrations of carbonyl groups (C=O) belonging to macromolecules. At the same time, the intense band at 730 cm^−1^ (double bond in the cis configuration and a long chain CH_2_) proves the presence of oleate fragments. The appearance of the broad band from the carboxylate ion of a very low intensity observed at 1610–1550 cm^−1^ may be attributed to the ionic form of the terminal group of **T1**, together with oleate fragments.

**Figure 8:**
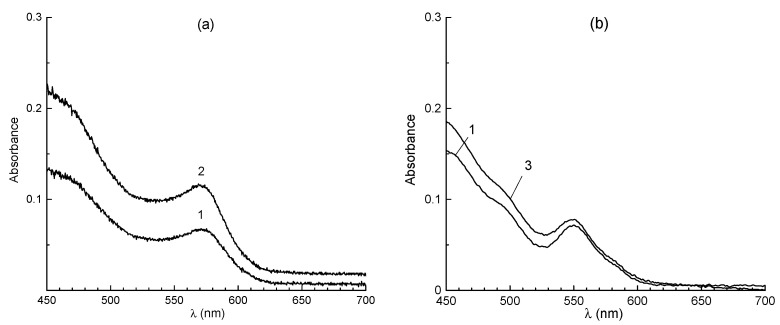
Absorption spectra of sols of nanocomposites (2.5 mg/mL) of PMMA with CdSe QDs (**a**) and CdSe/ZnS (**b**) in toluene: **M1** (1), and **T1** (2), **M2** (3).

Summarizing the data on morphology and molecular structure of complexes, we can see that if the content of QDs in a mixture with a polymer is about 1–3 wt%, the amount of the bound QDs is almost one order of magnitude lower. The weight fraction of CdSe QDs bound with monochelics is about twice higher than that of CdSe/ZnS QDs. However, the weight fraction of both QDs in nanocomposites with telechelics is higher than that in monochelics. The reason for the latter is the presence of two functional groups, which provide the interaction of macromolecules with QDs. This result conforms to the absorption spectral data. This trend is kept with the increase of MW of mono- and telechelics (Figure 8).

### 2.3. Optical Properties of Nanocomposites

**Figure 9:**
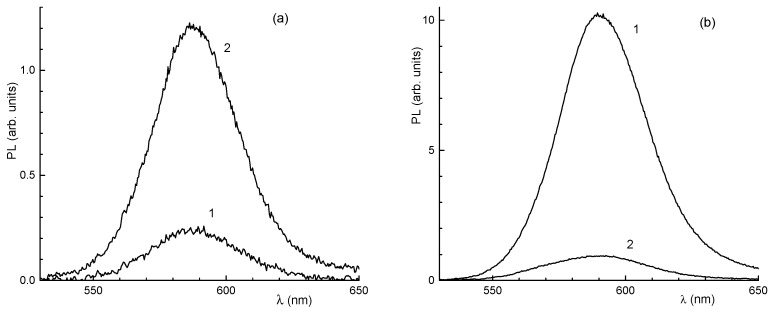
Photoluminescence spectra of sols of nanocomposites (2.5 mg/mL) in toluene based on CdSe QDs: (**a**) **M1** (1) and **M2** (2); (**b**) **T1** (1) and **T2** (2).

For photoluminescence measurements, nanocomposite sols based on PMMA chemically bound to the QDs surface via functional groups were used. As an example, Figure 9 shows the dependences of photoluminescence (PL) from the irradiation wavelength for QDs complexes with **M1**, **M2** (a), and with **T1** and **T2** (b).

As a whole, photoluminescence of nanocomposites (a) containing monochelic PMMA is relatively low due to lower amount of QDs, which is in agreement with absorption spectra data. Photoluminescence of nanocomposites based on telechelic PMMA is higher compared with nanocomposites based on monochelic PMMA. This phenomenon is related to the higher content of CdSe QDs due to the presence of two functional groups in telechelics, as mentioned above.

**Figure 10:**
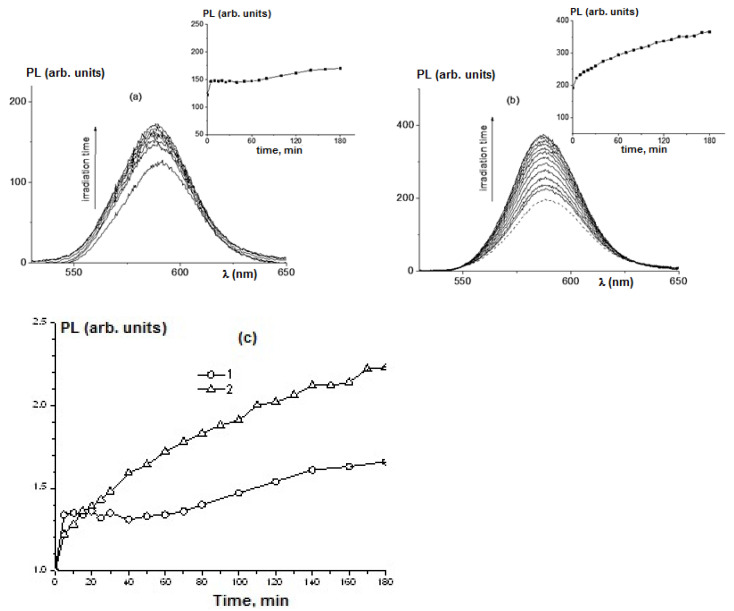
Photoluminescence spectra of sols of CdSe QDs stabilized by oleic acid (**a**) and **M1** (**b**) in toluene under continuous irradiation (λ_ex_ = 520 nm) for various times (from 0 to 180 min). The insets: Time dependence of the intensity of photoluminescence at λ = 576 nm for sols of CdSe QDs stabilized by oleic acid (1) and **M1** (2) in toluene under continuous irradiation; (**c**) normalized PL intensities for sols of CdSe QDs stabilized by oleic acid (1) and **M1** (2).

To compare the photostability of initial QDs with that of QDs with different stabilizers (or in different PMMA matrixes), we carried out the measurements of PL spectra at continuous irradiation. As an example, Figure 10 shows the spectra of PL of the initial CdSe QDs and their combination with M1 obtained at continuous laser irradiation with λ_ex_ = 520 nm. For more clarity, we add insets that show the dependence of PL_max_ on time. One can see the increase in PL with time. The growth of photoluminescence of quantum dots during continuous irradiation is known as photoactivation resulting from the rearrangement of QDs surfaces due to the light-induced heating contributed by the removal of defects [49,50]. This effect is much less pronounced in comparison with CdSe QDs stabilized by polystyrene [44]. However, the major result is that the continuous irradiation of nanocomposites with monochelic and telechelic does not lead to the drop of PL. It proves a very good photostability of CdSe QDs if they are stabilized by PMMA mono- and telechelics.

**Figure 11:**
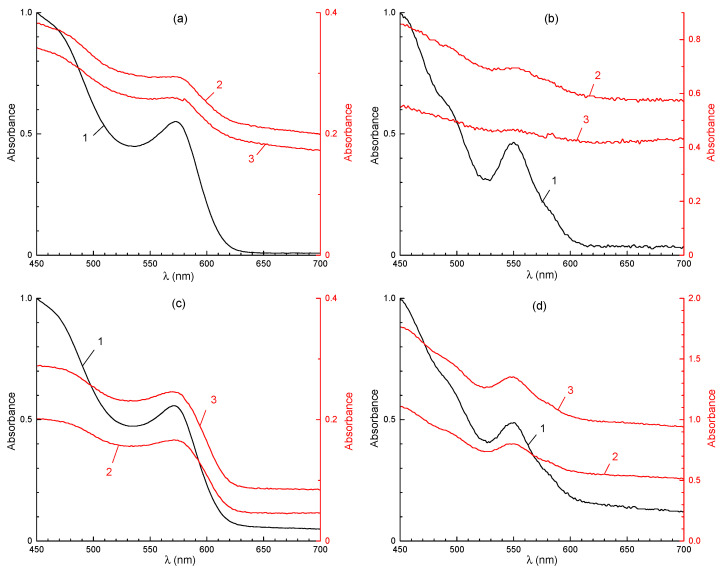
Absorption spectra for sols (1) and films before (2) and after annealing at 120 °C (3) of nanocomposites based on: **M1** (**a**,**b**) containing CdSe (**a**) and CdSe/ZnS QDs (**b**); **T1** (**c**,**d**) containing CdSe (**c**) and CdSe/ZnS QDs (**d**).

The last step was to compare the optical properties of QDs in sols and solid films before heating (1) and after it (2). The absorption spectra of sols (1) and solid films of CdSe (a,c) and CdSe/ZnS (b,d) with PMMA **M1** (a,b) and PMMA **T1** (c,d) are given as an example in Figure 11. The absorption maxima of CdSe QDs and CdSe/ZnS QDs are at 573 and 550 nm, respectively.

The peak maximum position of CdSe QDs with **M1** and **T1** in sols stays stable for both types of PMMA. The same behavior is seen for CdSe/ZnS, with the exception of annealed films, which show absorption that is too low.

Annealing of QDs results typically in the increase of the size of QDs and in the red-shift of the maximum in the photoluminescence spectrum (and exitonic peak in the absorption spectrum). This effect manifests itself most intensely in the sols of QDs [51].

**Figure 12:**
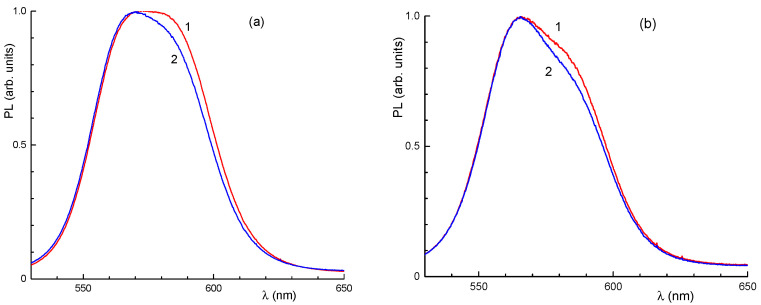
Photoluminescence spectra for films before (1) and after annealing at 120 °C (2) of CdSe/ZnS QDs and **M1** (**a**) and **T1** (**b**).

However, we did not observe the red-shift in our systems. Alternatively, annealing of QDs, in particular QDs with a core-shell structure, may lead to the rearrangement of the elements inside a QD and, as a consequence, to the shift of the photoluminescence and absorption spectra. It has been described in detail in [52] for ZnSe/ZnS core/shell quantum dots. As for PL maxima, they stay the same for CdSe and CdSe/ZnS QDs whether in **M1** or **T1** (Figure 12).

Thus, PMMA matrixes based on mono- or telechelics prevent aggregation of QDs even after annealing above glass transition temperature.

## 3. Materials and Methods

### 3.1. Materials

Methyl methacrylate (MMA), cadmium acetate, cadmium oxide, zinc acetate, oleic acid (99%), 1-octadecen (90%), sulfur, and selenium were supplied by Aldrich (St. Louis, MO, USA) and TOP (90%)—by Fluka (Buchs, Switzerland). MMA was distilled under reduced pressure before use. RAFT agents cyano-4-(phenylcarbonothioylthio)pentanoic acid (CPTPA) and *S*-(2-cyano-2-propyl)-*S*-dodecyl trithiocarbonate (CPDTC), initiator azobisisobutyronitrile (AIBN), sodium borohydride, and ethyl amine purchased from Aldrich were used as provided. Solvents benzene, DMF, CHCl_3_, THF, hexane, and 1,4-dioxane were distilled before use. Bidistilled toluene was used to prepare the solutions of PMMA and QDs.

### 3.2. Quantum Dots Synthesis

CdSe QDs were synthesized as described in [53] with the use of 1.5 mmol cadmium acetate and oleic acid (4.0 mmol) dissolved in 10 mL of 1-octadecen. The reaction mixture was heated at 160 °C for 1 h in a flow of argon to obtain cadmium oleate and to remove water. Then, the mixture was heated to a temperature of 180 °C, and a 1 mol/L selenium solution (1.5 mL) in TOP was added under intense stirring. After the growth of particles for 5 min, the reaction mixture was cooled to room temperature. QDs were precipitated via addition of acetone; they were centrifuged, washed twice with acetone, and dissolved in toluene. Obtained QDs presented CdSe non-spherical nanocrystals. According to TEM (Figure 6a), the resulting CdSe QDs were 3–5 nm in size.

CdSe/ZnS quantum dots were prepared with the use of cadmium oxide (0.4 mmol), zinc acetate (4 mmol), and oleic acid (17.6 mmol) as described in [54]. The above-mentioned reagents were mixed with 20 mL of 1-octadecene. The reaction mixture was gradually heated in a flow of argon under intense stirring until total dissolution. After that, the mixture was heated up to 280 °C, and the solution of 0.4 mmol of selenium and 4 mmol of sulfur powder dissolved in 3 mL of TOP was injected. Then, the reaction mixture was maintained at the high temperature for 5 min and rapidly cooled down to room temperature to stop the growth of QDs. QDs were precipitated out of reaction mixture in acetone. After that, we dissolved precipitated QDs in toluene and re-precipitated via addition of acetone. To purify QDs, the re-precipitation had to be done three times. According to TEM, the resulting core/shell sphere-like CdSe/ZnS particles were 4.5 ± 0.5 nm in size (Figure 6b).

### 3.3. Polymer Synthesis and Modification

PMMA was synthesized via RAFT polymerization according to the procedure described in [42]. Briefly, the required amount of AIBN and RAFT agent, CPTPA or CPTDC, was dissolved in MMA. Then the solution was poured into an ampoule, degassed, and sealed. After heating at 80 °C for 48 h, the reaction mixture was cooled and diluted with benzene; polymer was dried by lyophilization. Table 2 summarizes the conditions of the synthesis and molecular weight characteristics of the synthesized PMMA obtained by size-exclusion chromatography (SEC) and UV-vis spectrophotometry. In the latter case, molar mass was calculated based on the proposal that each macromolecule is living and contains a thiocarbonylthio end-group [55].

**Figure 13:**
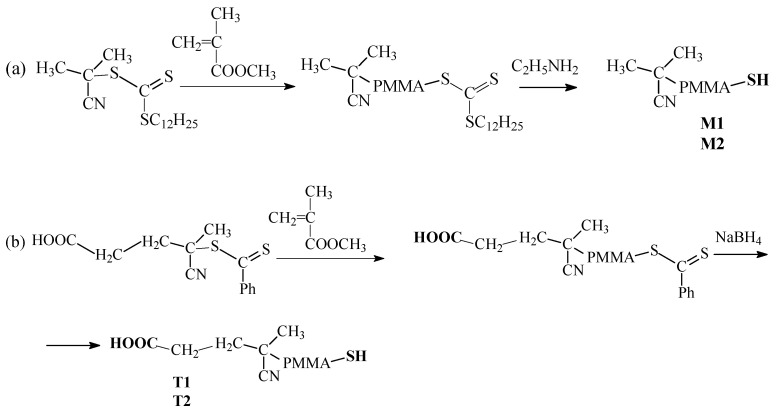
Synthetic route and designation of monochelic PMMA (**a**) and telechelics of PMMA (**b**).

Thiol-terminated PMMA was prepared by reduction of dithiobenzoate end-group with sodium borohydride or by aminolysis of trithiocarnonate end-group with ethyl amine as described in [42]. The completeness of the reaction was confirmed by UV-vis spectrophotometry by absence of the absorbance in the range of 300–310 nm. Table 3 summarizes the molecular weight characteristics of the modified PMMA, and Figure 13 represents the synthetic route and chemical structure of the samples. We defined thiol-terminated PMMA as monochelic (**M1**, **M2**) and PMMA containing both carboxyl and thiol end-groups as telechelic (**T1** and **T2**).

### 3.4. Preparation of PMMA—QDs Nanocomposite Films and Sols

To prepare the nanocomposite, mono- or telechelic PMMA were dissolved in freshly distilled toluene (5 mg/mL). To this solution, the required amount of the solution of QDs in toluene was added. Prepared homogeneous mixture of slightly red color was left for 3–4 days without stirring. Obtained solutions were used for DLS analysis and TEM. For the analysis of absorption and PL spectra, prepared mixtures were precipitated in the excess of hexane and centrifuged to remove free QDs. After that, the nanocomposite was dissolved in toluene and dried by liophilization in vacuum.

### 3.5. Instrumentation

The average molecular weights and dispersity (Ð) were determined by SEC. The SEC measurements were performed in DMF containing 0.1 wt% of LiBr at 50 °C with a flow rate of 1.0 mL min^−1^ using a chromatograph PolymerLabs GPC-120 equipped with refractive index detector and with two columns PLgel 5 µm MIXED B for MW range 5 × 10^2^–1 × 10^7^. The SEC system was calibrated using narrow dispersed linear poly(methyl methacrylate) with MW ranging from 800 to 2 × 10^6^ g mol^−1^.

The concentration of thiocarbonylthio end-groups in RAFT-based PMMA was measured in THF on a Unico 2804 UV-vis spectrometer (UNICO, Fairfield, NJ, USA) according to the procedure described in [55].

The absorbance spectra of QDs and composites were measured with a spectrometer Ocean Optics USB 2000 (Ocean Optics, Dunedin, FL, USA) and halogen lamp light source. Spectra were measured by Ocean Optics SpectraSuite software (Ocean Optics, Dunedin, FL, USA). The spectrum of the quartz cell with pure chloroform was used as a reference.

The PL spectra of QDs and nanocomposites with PMMA in sols were recorded using a spectrometer Ocean Optics USB 2000 in geometry close to 90°, using sealed glass optical cells. Solutions composites in “dry” toluene were blown with dry argon. Laser module with wavelengths of 520 nm and radiation power of 10 mW were used as excitation source. Spectra were measured by Ocean Optics SpectraSuite software. All components in measuring the systems were isolated from light.

PL spectra of solid films were recorded using Solar Laser Systems M266 automated monochromator/spectrograph (Minsk, Belarus) equipped with the spectroscopic multi-channel detector Ormins U2C–16H7317 (Minsk, Belarus), a homemade light-collecting inverted system using Olympus MPLAPON 100X/0.95 objective (Tokio, Japan), and a homemade confocal unit with two 100 mm lenses. Exciting light was cut off by Semrock 488 nm RazorEdge^®^ ultrasteep longpass edge filters (Rochester, NY, USA). Fluorescence of QDs was excited by an KLM-473/h 150 laser (Plazma, Moscow, Russia) operating at 473 nm. An incident light intensity was equal to 50 mW/cm^2^ as measured with Coherent LaserMate-Q intensity meter [56].

TEM images were obtained on a transmission electron microscope Carl Zeiss LEO 912 AB OMEGA (Oberkochen, Germany) operating at 100 kV accelerating voltage. Samples were drop cast from toluene sol onto Formvar^®^-coated copper grids (Polysciences, Warrington, PA, USA).

The interaction of end-functionalized PMMA with QDs was studied by DLS. All the measurements were performed on multi-angle particle size analyzer Photocor Complex (Photocor Instruments, Moscow, Russia). A 10 mW He-Ne laser (λ = 633 nm, Moscow, Russia) was used as a light source. Toluene and analyzing solutions were filtered using 0.45 μm Millipore filters (Aldrich). The time correlation function of the scattered light intensity fluctuation g_2_(τ) was measured by digital real-time 288 channel multi-tau correlator Photocor-FC (Photocor Instruments, Moscow, Russia), equipped with pseudo-cross-correlative photon counter (Photocor Instruments, Moscow, Russia). Data processing was performed using Photocor Instruments DynaLS software using the following equation: g_2_(τ) = B + βexp(−2Γτ), where B is the baseline of the correlation function at infinite delay, β is the correlation function amplitude at zero delay, τ is the delay time, and Γ is the decay rate. Correlation functions were then used to determine z-mean values of the diffusion coefficient and the distribution of the diffusion coefficients using cumulant analysis and regularization procedure, respectively. Hydrodynamic radii of the particles were then calculated using the Stokes–Einstein equation: R_h_ = kT/6πη_0_D, where R_h_ is the hydrodynamic radius, k = 1.38 × 10^23^ J/K—the Boltzmann constant, T is temperature, η_0_ is the viscosity of the solvent, and D is the diffusion coefficient.

## 4. Conclusions

The present research addresses the problem of preparation of luminescent nanocomposites based on QDs embedded in the matrix of end-functionalized PMMA. This approach enables QDs to keep their optical properties and to control the space between QDs. End-functionalization is provided by RAFT polymerization, which is responsible for the synthesis of macromolecules with precise chain architecture. It is clear from general consideration that the ability of the polymeric chain to attach to the surface of a QD is determined by the number of end-functional groups per chain and its molecular weight. Hence, it was reasonable to assume that telechelic PMMA should be more promising than monochelic PMMA as a stabilizer and the matrix for QDs.

This assumption was confirmed experimentally by DLS, TEM, and photoluminescence, as well as by absorbance data. The better binding of telechelic PMMA to QD than monochelic PMMA provides higher PL of the nanocomposite after purification from ‘free’ QDs.

In both cases, we observed the fluorescence emission after annealing of the films and good photostability of QDs stabilized by end-functionalized PMMA after the continuous irradiation.

## Figures and Tables

**Table 1:** The content of QDs in nanocomposites with monochelic PMMA (**M1** and **M2**) and telechelic PMMA (**T1** and **T2**).

QDs	Sample	Weight Fraction of QDs in Mixture, %	Weight Fraction of QDs in Nanocomposite, %
CdSe	**M1**—QDs	1.4	0.39
	**M2**—QDs	1.4	0.67
	**T1**—QDs	1.4	1.13
	**T2**—QDs	1.4	0.78
CdSe/ZnS	**M1**—QDs	3.2	0.16
	**M2**—QDs	3.2	0.22
	**T1**—QDs	3.2	1.12
	**T2**—QDs	3.2	0.86

**Table 2:** Experimental conditions for PMMA synthesis.

Sample	RAFT Agent, mol/L	AIBN, mol/L	Conversion, %	M_n_, kDa (SEC)	Ð	M_n_, kDa (UV)
PMMA1	CPTPA, 7 × 10^−2^	1 × 10^−3^	33.4	9.6	1.11	10.0
PMMA2	CPTPA, 3.2 × 10^−2^	5 × 10^−4^	32.0	28.1	1.16	28.4
PMMA3	CPDTC, 3 × 10^−2^	5 × 10^−4^	55.1	7.7	1.23	8.0
PMMA4	CPDTC, 5 × 10^−2^	1 × 10^−4^	38.0	33.4	1.22	35.2

**Table 3:** Molecular weight characteristics of mono- and telechelic PMMA.

Sample	End Functionality	RAFT Agent	Modification	M_n_, kDa	Đ
**T1**	SH and COOH	CPTPA	NaBH4	9.6	1.11
**T2**	SH and COOH	CPTPA	NaBH4	28.1	1.16
**M1**	SH	CPDTC	C2H5NH2	7.7	1.23
**M2**	SH	CPDTC	C2H5NH2	33.4	1.22

## Data Availability

The raw/processed data required to reproduce these findings cannot be shared at this time as the data also forms part of an ongoing study.

## Sample Availability

No samples are available from the authors.
